# Analysis of intestinal flora and cognitive function in maintenance hemodialysis patients using combined 16S ribosome DNA and shotgun metagenome sequencing

**DOI:** 10.1007/s40520-023-02645-y

**Published:** 2024-02-09

**Authors:** Qiuyi Gao, Dianshi Li, Yue Wang, Chunhui Zhao, Mingshuai Li, Jingwen Xiao, Yan Kang, Hongli Lin, Nan Wang

**Affiliations:** 1 Department of Nephrology, The First Affiliated Hospital of Dalian Medical University, Dalian, China; 2 Centre for Empirical Legal Studies, Faculty of Law, University of Macau, Macau, China; 3 Department of Nephrology, Binzhou Medical University Affiliated Shengli Oilfield Central Hospital, Binzhou, China; 4 School of Graduate, Dalian Medical University, Dalian, China

**Keywords:** Cognitive function, Intestinal flora, Microbiota, Maintenance dialysis, 16S rDNA, Shotgun metagenome sequencing

## Abstract

**Background:**

Cognitive impairment is widely prevalent in maintenance hemodialysis (MHD) patients, and seriously affects their quality of life. The intestinal flora likely regulates cognitive function, but studies on cognitive impairment and intestinal flora in MHD patients are lacking.

**Methods:**

MHD patients (36) and healthy volunteers (18) were evaluated using the Montreal Cognitive Function Scale, basic clinical data, and 16S ribosome DNA (rDNA) sequencing. Twenty MHD patients and ten healthy volunteers were randomly selected for shotgun metagenomic analysis to explore potential metabolic pathways of intestinal flora. Both16S rDNA sequencing and shotgun metagenomic sequencing were conducted on fecal samples.

**Results:**

*Roseburia* were significantly reduced in the MHD group based on both 16S rDNA and shotgun metagenomic sequencing analyses. *Faecalibacterium*, *Megamonas*, *Bifidobacterium*, *Parabacteroides*, *Collinsella*, *Tyzzerella*, and *Phascolarctobacterium* were positively correlated with cognitive function or cognitive domains. Enriched Kyoto Encyclopedia of Genes and Genomes (KEGG) pathways included oxidative phosphorylation, photosynthesis, retrograde endocannabinoid signaling, flagellar assembly, and riboflavin metabolism.

**Conclusion:**

Among the microbiota, *Roseburia* may be important in MHD patients. We demonstrated a correlation between bacterial genera and cognitive function, and propose possible mechanisms.

**Supplementary Information:**

The online version contains supplementary material available at 10.1007/s40520-023-02645-y.

## Introduction

As a result of improved dialysis techniques and reduced mortality, the number and lifespan of patients undergoing maintenance hemodialysis (MHD) is growing globally [1, 2]. However, the quality of life (Qol) for MHD patients is less than satisfactory. Mild cognitive impairment has an adverse effect on Qol, while severe cases can lead to disability and an increasingly heavy healthcare burden [3]. Cognitive impairment is diagnosed when cognitive functions decline in at least one or more cognitive domains, including attention, memory, language, comprehension and judgment, executive function, and others. It can range from mild forms of forgetfulness to severe dementia [4]. As the incidence of chronic kidney disease increases, the degree of cognitive impairment becomes more severe. Cognitive impairment incidence for MHD is 30–60.9%, which is twice the incidence in the age-matched general population [5–8]. Diagnosis of mild cognitive impairment (MCI, a transitional state with minimal cognitive impairment and relatively intact daily functioning) or Alzheimer’s disease (AD) is mainly based on clinical manifestations [9]. However, the mechanisms of cognitive impairment in MHD patients remain unclear. Besides genetic factors, non-genetic related factors play an important role in the development of cognitive dysfunction. These include decreased cerebral blood flow caused by chronic kidney disease (CKD)-related vascular factors (such as atherosclerosis), glymphatic fluid transport issues caused by endothelial dysfunction, and uremic toxin factors that cannot be excreted from the body because of renal damage. These can result in neuronal damage and lead to cognitive dysfunction.

The gut microbiota is known to participate in substance synthesis, nutrient absorption, regulation of immunity, and preventing infection [10, 11]. In recent years, the microbiota–gut–brain axis theory has gained wide acceptance. This theory describes the bidirectional connection between the microbiota and psychiatric disorders through nervous, endocrine, and immune mechanisms. Also, a change in the composition of the gut microbiota occurs in patients with CKD or MHD, including an increase in potentially pathogenic species and a decrease in beneficial species [12]. However, to our knowledge, research on the relationship between the microbiota and cognitive function in MHD patients has not been reported.

Both 16S rDNA and shotgun metagenome sequencing are widely employed to investigate the microbiota. These methods are based on high-throughput sequencing technology, but they are quite different in principle, purpose, sequencing depth, and cost. Specifically, 16S rDNA sequencing involves PCR amplification of specific hypervariable regions, which is cheaper and widely used to analyze bacterial diversity [13–15]. By contrast, shotgun metagenomic sequencing amplifies all microbial genomic DNA, then assembles genomic fragments for gene prediction [16]. Herein, we employed a combined approach to evaluate the cognitive functions of MHD patients in our clinic. First, 16S rDNA sequencing technology was used to characterize the microbiota in MHD patients. Next, the microbiota was correlated with cognitive function. Finally, shotgun metagenome sequencing was used to identify the functional pathways potentially involved.

## Materials and methods

**Fig. 1:**
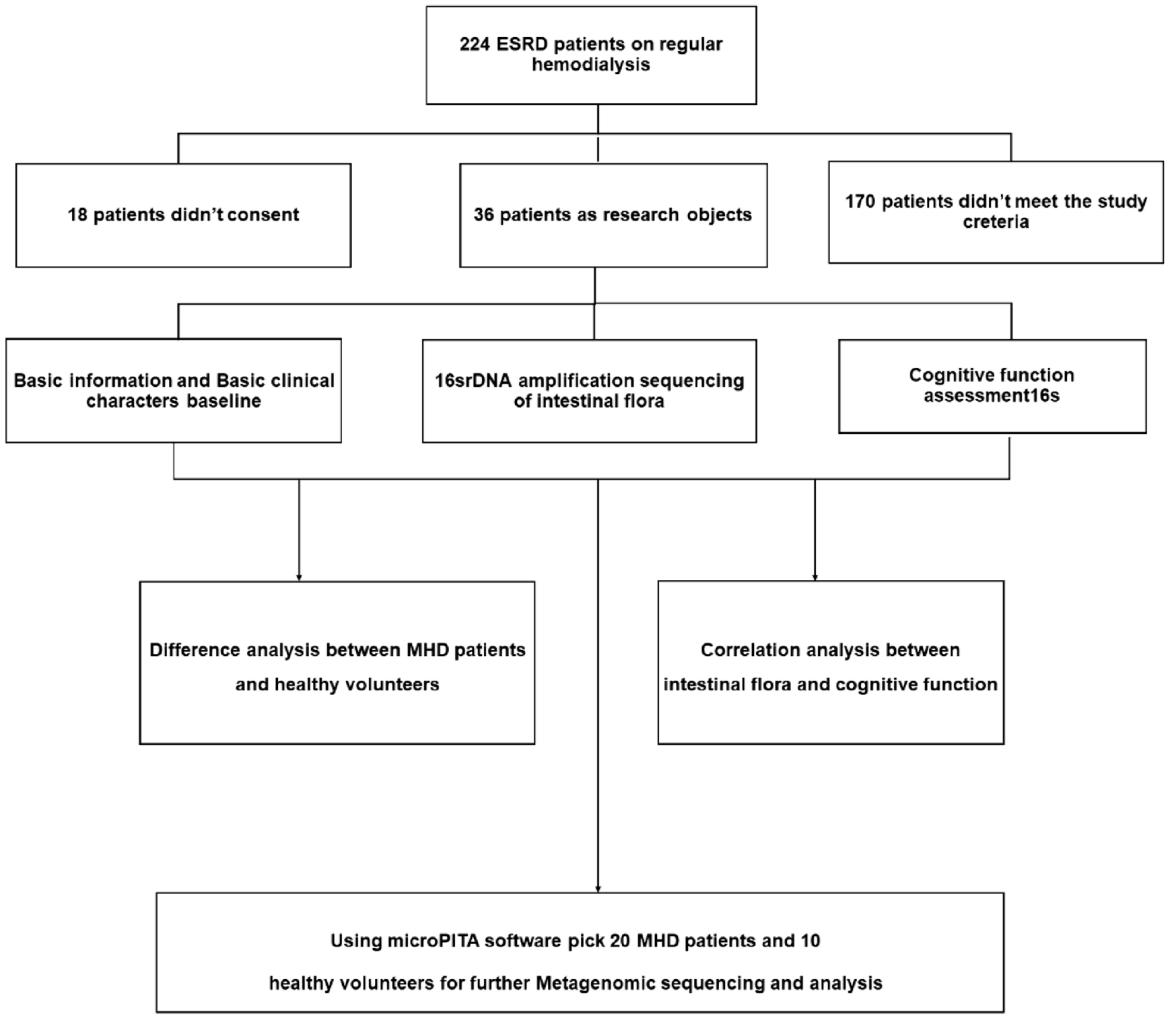
Basic experimental scheme

In our cross-sectional study, 36 dialysis patients from the Blood Purification Center of the First Affiliated Hospital of Dalian Medical University were included, all of whom gave informed consent form and agreed to participate. This work took place from December 2018 to January 2019. To eliminate the endogenous differences caused by age and sex at maximum extent, we matched 18 normal persons to the 36 patients at a 1:2 ratio. The basic experimental scheme is shown in Fig. 1.

### Criteria for patient recruitment

The inclusion criteria for the MHD group met the KDIGO criteria of CKD stage 5; estimated glomerular filtration rate (eGFR) < 15 ml/(min‧1.73m^2^), and regular hemodialysis at least three times a week for more than 3 months. The exclusion criteria were as follows: (1) patients with eating or digestive system disorders; (2) patients with cerebrovascular disease (serious cerebral infarction, cerebral hemorrhage), Parkinson’s disease (PD), depression, autism, or a history of substance abuse; (3) patients who had taken antibiotics, immunosuppressants, or glucocorticoids in the past 3 months; (4) patients suffering from a life-threatening disease.

Eighteen sex-, age-, and education-matched healthy volunteers who were not taking any medication and who had not previously reported any other disease were recruited (hemodialysis: healthy = 2:1). All participants gave written informed consent. This research protocol passed the ethical review of the Ethics Committee of Dalian Medical University (ethics number PJ-KS-KY-2018-74).

### Clinic data collection and evaluation of cognitive function

We collected basic data including height, weight, age, gender, education level, systolic blood pressure, and diastolic blood pressure for all enrolled patients. MHD patients also included information on primary disease, duration of dialysis, frequency of dialysis, and adequacy of dialysis. All participants were required to maintain normal eating habits, and blood samples were taken following fasting for more than 8 h. Blood samples were immediately stored at 0–4 degrees and tested by the Department of Clinical Laboratory of the First Affiliated Hospital of Dalian Medical University for glucose, lipids, renal function, electrolyte, and high-sensitivity C-reactive protein. On the same day, all participants were analyzed using the Montreal Cognitive Assessment (MoCA) scale by the same trained operator following the operating instructions before receiving hemodialysis treatment. The maximum score is 30, and 26 was considered the cut-off value (i.e., a score < 26 was considered to indicate cognitive dysfunction, while > 26 was considered to indicate normal cognitive function). MHD patients underwent cognitive function evaluation 2 h before hemodialysis. We collected feces from participants 2 days before blood collection to ensure the reliability of the results. Feces were collected by multi-point sampling, stored on ice throughout transportation, and sent for 16S rDNA and metagenomic sequence analysis.

### 16S rDNA amplifiers and shotgun metagenomic sequencing

We performed 16S rDNA amplification for all participants. We used microPITA (Microbiomes: Picking Interesting Taxa for Analysis) [17] software in a representative way to automatically and randomly generate the selected sample IDs for shotgun metagenomic sequencing (20 dialysis patients and 10 healthy people). Beijing Novogene Technology Co., Ltd (Beijing, China) performed sequencing and analysis of 16S rDNA and shotgun metagenomic sequencing data from fecal samples. Details of 16S rDNA and shotgun metagenomic sequencing including DNA extraction, PCR amplification, sequencing and bioinformatics analysis can be found in the additional material. Row data used in the study are publicly available. The 16r DNA sequencing data can be found at [https://www.ncbi.nlm.nih.gov/sra?LinkName=biosample_sra&from_uid=29625227 under accession code BioProject PRJNA860849. The shotgun metagenomic sequencing data can be found at [https://www.ncbi.nlm.nih.gov/bioproject/PRJNA849628 under accession code BioProject PRJNA849628.

### Statistical analyses

We analyzed differences in general conditions, cognitive function scale scores, and laboratory indices between dialysis patient and healthy groups. The Kolmogorov–Smirnov *Z* test was used to assess the normality of quantitative data. When the data obeyed a normal distribution and the variance was uniform, two independent sample *T* test was used, otherwise Mann–Whitney non-parametric test was used. For the microbiota, *T* test was used to analyze differences in alpha-diversity between the two groups, while differences in beta-diversity were analyzed by unweighted Wilcoxon test. Linear discriminant analysis effect size (LEfSe) was used to identify meaningful biomarkers with statistical differences (LEfSe < 4) between groups. Spearman correlation analysis was performed between the 35 most abundant intestinal microflora components of MHD patients and the general condition or Montreal Cognitive Assessment (MoCA) scale (*p* < 0.05). Intestinal flora data were analyzed using R (Version2.15.3, the R software can be found at https://www.r-project.org/) and other data were analyzed by the software of Statistical Package for Social Sciences 22.0.

## Results

### Basic clinical characteristics and MoCA scoring of cognitive function

A total of 36 MHD patients and 18 healthy volunteers were recruited, and there were no statistical differences between the 2 groups in terms of gender, age, height, weight, education level, and diastolic blood pressure. However, the systolic pressure (SP) was higher in MHD patients (*p* < 0.05). MHD patients had a higher prevalence of cognitive dysfunction (61% vs. 28% in healthy volunteers, *p* < 0.05), and the difference was mainly manifested in the total MoCA score, visual space/executive function, naming, and language domains (all *p* < 0.05), but there were no differences in attention, abstract ability, memory or orientation. Further details are shown in Table 1.

**Table 1:** Basic clinical characteristics and MoCA scores for cognitive function

Parameters (units)	MHD group (*n* = 36)	Healthy group (*n* = 18)	Statistical value (*T* or *Z* value)	Significance (double tail)
Gender (male/female)	(18/18)	(9/9)	0.00	1
Age (years)	61.06 ± 11.42	60.28 ± 7.06	0.26	0.793
Height (cm)	168 ± 7.87	167.44 ± 5.55	0.27	0.79
Weight (kg)	67.74 ± 11.06	69.35 ± 9.16	0.53	0.596
Education level	3.5 (2, 5)	3.5 (2, 5)	1.23	0.218
Systolic blood pressure (mmHg)	147.5 (130,155)	120 (119.25, 130)	4.27	0.000^**^
Diastolic blood pressure (mmHg)	80 (80, 90)	80 (77.75, 81.25)	1.47	0.143
*Kt/v*	1.35 ± 0.79	–	–	–
Dialysis age (years)	5.15 ± 4.12	–	–	–
Primary disease				
Primary glomerulonephritis	23 (63.9%)	–	–	–
Hypertensive renal damage	6 (16.67%)	–	–	–
Diabetic nephropathy	4 (11.1%)	–	–	–
Polycystic kidney	2 (5.5%)	–	–	–
Other	1 (2.8%)	–	–	–
Total Montreal Cognitive Scale (MoCA) score	25 (23, 26.75)	27.5 (24.75, 28)	2.03	0.042*
Percentage of cognitive impairment (normal/abnormal)	61% (14/22)	28% (13/5)	–	–
Visual space/executive function	3 (3, 4)	4.5 (3, 5)	2.23	0.026^*^
Naming	3 (2, 3)	3 (3, 3)	2.07	0.038^*^
Attention	6 (5, 6)	6 (5, 6)	0.03	0.974
Language	1.5 (1, 2)	2 (1, 3)	2.14	0.033^*^
Abstract ability	2 (1, 2)	2 (1, 2)	0.38	0.701
Memory	4 (3, 5)	4 (4, 5)	1.27	0.205
Orientation	6 (6, 6)	6 (6, 6)	0.28	0.777

** and * represent differences between MHD and healthy groups at *p* < 0.01 and *p* < 0.05

Education level (1 = primary school, 2 = junior high school, 3 = senior high school, 4 = junior college, 5 = undergraduate)

### 16S rDNA sequencing

A total of 2 kingdoms, 21 phyla, 34 classes, 70 orders, 130 families, 283 genera, and 266 species were identified by the 16S rDNA technique.

#### Alpha–beta-diversity analysis

**Fig. 2:**
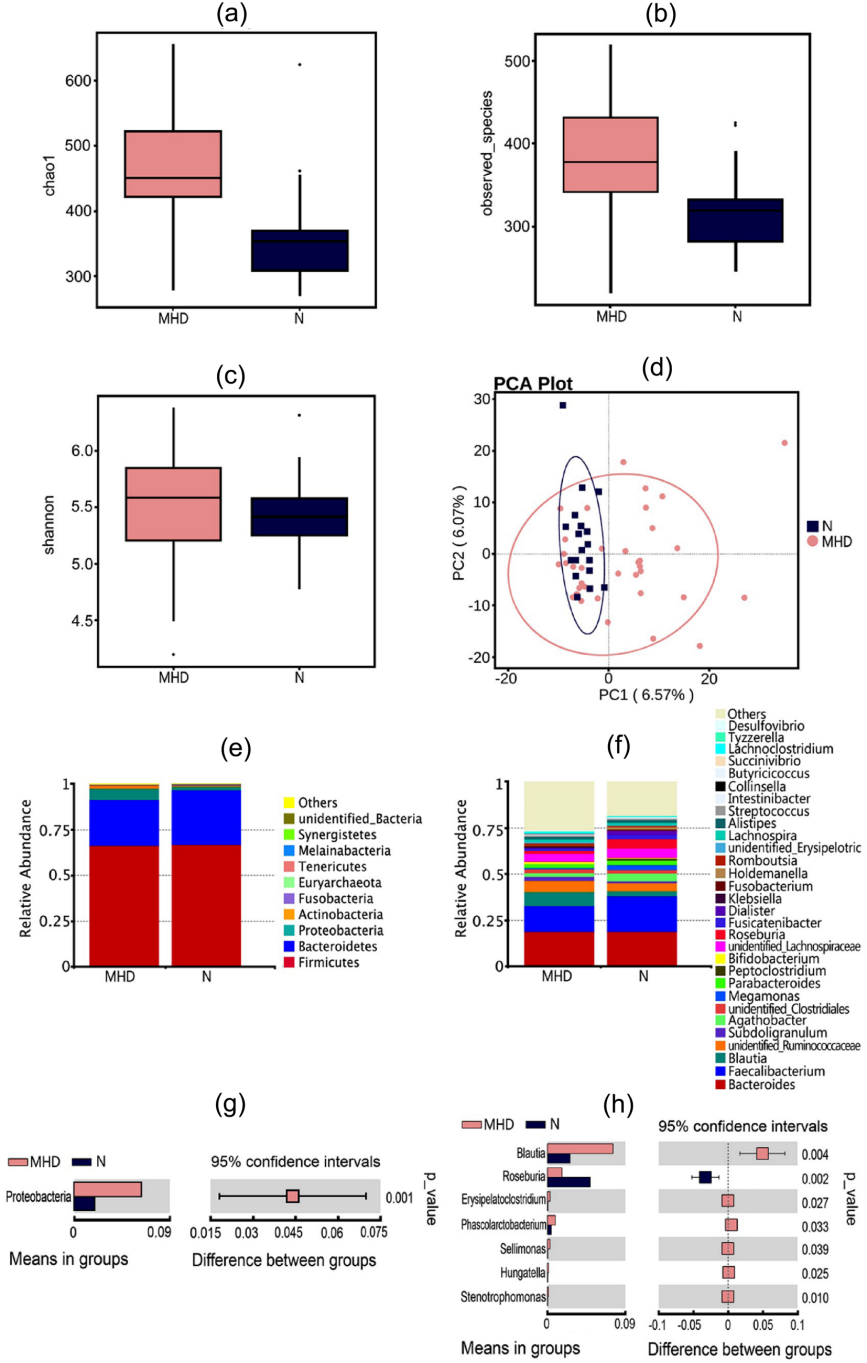
Analysis of differences in the microbiota based on 16S ribosome DNA (rDNA) sequencing between MHD patients and healthy people. **a**–**c** Chao1, Observed_species. and Shannon indices, respectively. Chao1 and Observed_species explain α-diversity (within-habitat diversity), and both indices were significantly increased in MHD patients (Figure **a** and **b**, *p* = 0.000 and 0.001, respectively). Meanwhile, the Shannon index **c** showed no significant difference between the two groups (*p* = 0.541). **d** Principal component analysis (PCA) of differences in the composition of the intestinal flora. The principal component contribution values of horizontal and vertical axes are 6.57% and 6.07%, respectively. **e** and **f** The main components of the microbiota at phylum and genus levels, respectively. **g** and **h** Differences in the microbiota between the two groups at phylum and genus level, respectively

Chao1, Observed_species, and Shannon index were calculated based on different principles for α-diversity (within-habitat diversity) evaluation of the microbiota [18]. The results showed that Chao1 and Observed_species indices were significantly increased in MHD patients (Fig. 2a and b, *p* = 0.000 and 0.001, respectively). Meanwhile, the Shannon index, which reflects species richness and evenness, showed no significant difference (*p* = 0.54) in community diversity between MHD patient and healthy groups (Fig. 2c). We performed principal component analysis (PCA) based on variance decomposition, and selected the largest contribution rate for graph plotting. The principal component contribution values for horizontal and vertical axes were 6.57% and 6.07%, respectively (Fig. 2d). The results revealed huge inter-individual differences for the horizontal axes (composition of the intestinal flora) between MHD and healthy groups.

#### Relative abundance and diversity of the microbiota

A total of one phylum, two classes, three orders, seven families, seven genera, and eight species were identified from *T *test statistical analysis of the relative abundances of microbiota components between the two groups (all *p* < 0.05). *Firmicutes*, *Bacteroidetes*, *Proteobacteria*, and *Actinobacteria* were the main components, and their relative abundances decreased successively (Fig. 2e and f). Compared with the healthy group, the relative abundance of the *Proteobacteria* phylum was highest in the MHD group (*p* < 0.01, Fig. 2g). At the genus level, *Bacteroides* was dominant in both groups. *Roseburia* was significantly reduced in the MHD group, while *Blautia*, *Erysipelatoclostridium*, *Phascolarctobacterium*, *Sellimonas*, *Hungatella*, and *Stenotrophomonas* were increased significantly (all *p* < 0.01; Fig. 2h).

##### Analysis of correlations between the microbiota and cognitive function

**Fig. 3:**
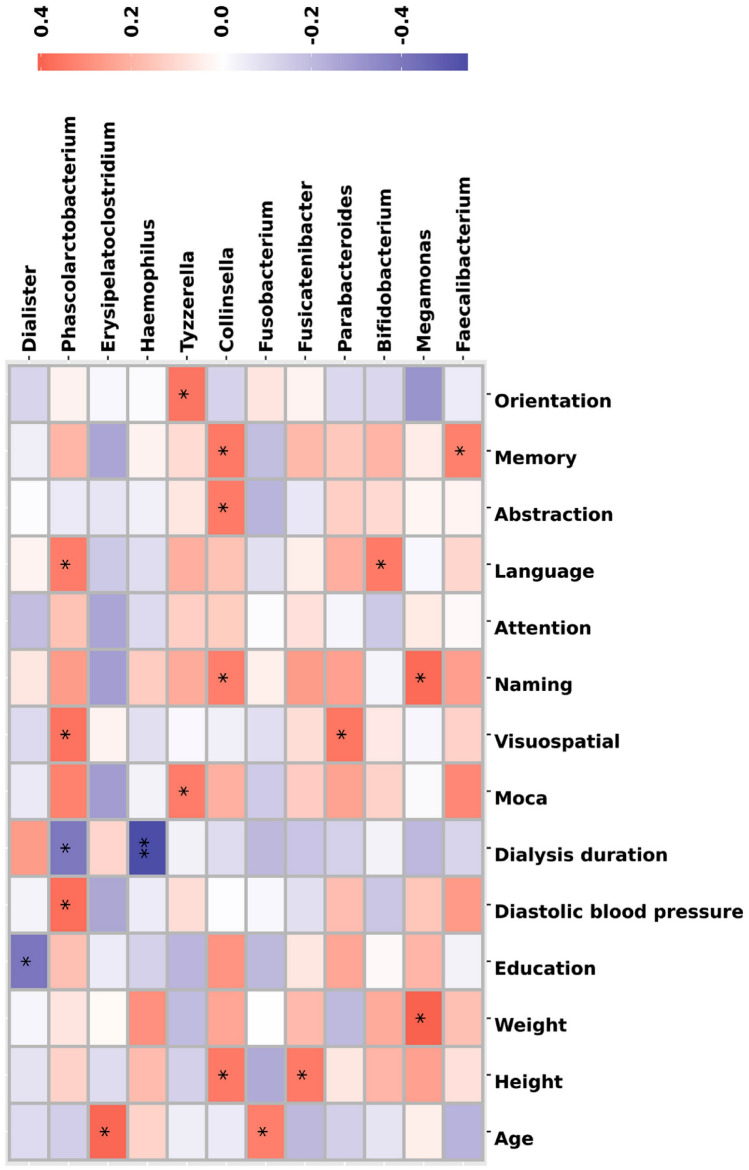
Spearman correlation analysis between the microbiota and cognitive function. Twelve of the top thirty-five bacterial genera show a significant positive correlation with cognitive function or cognitive domains (all *p* < 0.05)

A spearman correlation analysis between the top 35 most abundant bacterial genera, general conditions, and cognitive function was conducted on all MHD patients (*n* = 36). A total of 12 bacterial genera showed a significant correlation, among which *Faecalibacterium*, *Megamonas*, *Bifidobacterium*, *Parabacteroides*, *Collinsella*, *Tyzzerella*, and *Phascolarctobacterium* were positively correlated with cognitive function or cognitive domains (all *p* < 0.05). *Phascolarctobacterium* showed a positive correlation with visual space/executive power (r = 0.37, *p* = 0.027; Fig. 3) and language function (*r* = 0.34, *p* = 0.042; Fig. 3), but showed a negative correlation with the duration of dialysis (*r* = − 0.40, *p* = 0.018; Fig. 3). *Erysipelatoclostridium* was positively correlated with age (*r* = 0.39, *p* = 0.018; Fig. 3).

### Shotgun metagenomic sequencing

In our study, 20 MHD patients and 10 healthy people were randomly selected by microPITA [17] to explore functional genes and metabolic pathways. A comparison of taxonomic annotation and functional genes was performed between MHD patients and healthy people.

#### Taxonomic annotation of shotgun metagenomic sequencing data

**Fig. 4:**
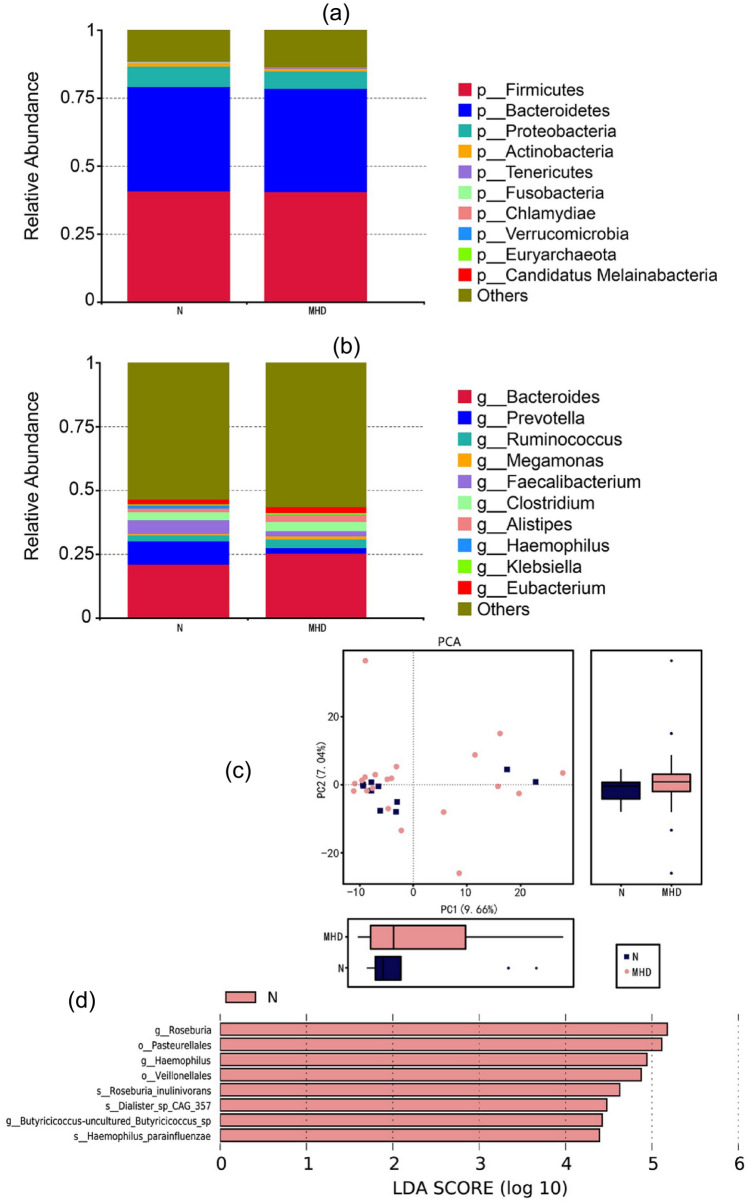
Differences in α-diversity and β-diversity between MHD patients and healthy people based on metagenomic sequencing. **a** and **b** The main components of the microbiota at phylum and genus levels, respectively. *Firmicutes, Bacteroidetes, Proteobacteria, Actinobacteria* and *Bacteroides* are the main components of the microbiota. **c** PCA of differences in intestinal flora between MHD and healthy control groups. The principal component contribution values of horizontal and vertical axes are 9.66% and 7.04%, respectively. **d** *Roseburia_inulinivorans*, *uncultured_Butyricicoccus_sp, Dialister_sp_CAG_357* and *Haemophilus_parainfluenzae* are the most abundant component of the intestinal microbiota in the healthy group based on linear discriminant analysis effect size (LEfSe)

Figure 4a and b shows the relative abundance of intestinal flora using shotgun metagenomic sequencing technology at phylum and genus levels, respectively. *Firmicutes, Bacteroidetes*, *Proteobacteria*, and *Actinobacteria* remain the main bacterial flora components. At the genus level, *Bacteroides*, *Prevotella*, *Ruminococcus*, *Megamonas*, and Faecalibacterium constitute the main components of the intestinal flora.

We performed PCA on the shotgun metagenomic sequencing data annotation results and selected the main contributors for graphical display. At the phylum level, there was a difference in the composition of the intestinal flora between MHD and healthy groups, and the principal component contribution values for the horizontal and vertical axes were 9.66% and 7.04%, respectively (Fig. 4c).

LEfSe is an efficient tool for finding biomarkers with statistical differences between groups, and it works by combining parametric and non-parametric testing. Our results showed that the relative abundance of *Roseburia_inulinivorans*, *uncultured_Butyricicoccus_sp*, and *Dialister_sp_CAG_357* belonging to *Firmicutes*, and *Haemophilus_parainfluenzae* belonging to *Proteobacteria*, was more abundant in the healthy group (Fig. 4d).

#### KEGG pathway function annotation of shotgun metagenomic sequencing data

**Fig. 5:**
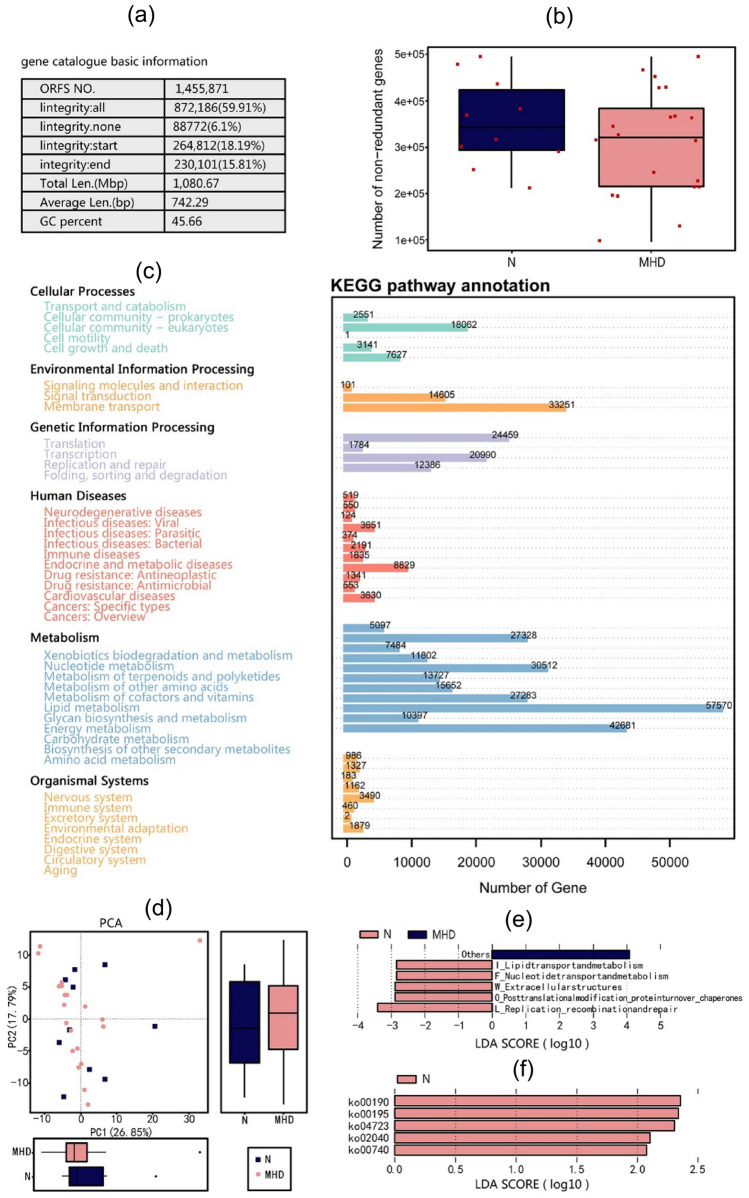
KEGG pathway function annotation of metagenomic sequencing data. **a** Basic statistical information for metagenomic sequencing analysis. **b** Box plot showing fewer functional genes in the MHD group. **c** Statistical analysis of the number of KEGG unigene annotations. The numbers on the bar chart represent the number of unigenes on the annotations, and the other axis lists the codes for each level 1 functional class in each database. **d** PCA showing differences in KEGG pathway function annotation between MHD and healthy groups. **e** and **f** Differences in KEGG pathways between the two groups at KEGG pathway levels 2 and 3, respectively

Figure 5a shows an overview of the results of functional gene annotation. The box plot shows the differences in the number of genes between MHD and healthy groups. The MHD group had fewer functional genes (Fig. 5b). Figure 5c shows an overview of the results of KEGG pathway function annotation. The results reveal differences in functional classification, and the numbers on the bar graph represent the number of unigenes for the annotation, while the other axis lists the codes for each level 1 class in the KEGG pathway database. PCA revealed differences in KEGG pathway function annotation between MHD and healthy groups (Fig. 5d). Functional genes related to lipid transport and metabolism, nucleotide transport and metabolism, extracellular structures, posttranslational modification, protein turnover, chaperones, and replication-recombination and repair were enriched in the healthy group (Fig. 5e). Genes associated with the ko00190 (oxidative phosphorylation), ko00195 (photosynthesis), ko04723 (retrograde endocannabinoid signaling), ko02040 (flagellar assembly), and ko00740 (riboflavin metabolism) were especially enriched (Fig. 5f).

Of the 20 MHD patients, 15 were cognitively abnormal (HD.UN) and 5 were cognitively normal (HD.N). We conducted differential metagenomic analysis between the two groups, and Chao1, Observed_species, and Shannon index showed a significant increase in MHD.N patients (Online Resource 1 a, b, c). LEfSe analysis revealed that the relative abundances of S_*Megamonas_funiformis*, 0_*Verrucomicrobiales*, and f_*Akkermansiaceae* were higher in the HD.UN group. Meanwhile, S_ *Sutterella_ wadsworthensis*, S_*Bacteroidales_bacterium_43_36*, g_*Chryseobacterium*, S_*Azobacteroides_phage_PrpJPt_Bp1*, S__*Porphyromonadaceae_bacterium_KH3CP3RA*, g_*Sutterella*, and S_*Veillonella_sp_CAG_933* were elevated in the HD. N group (Online Resource Fig. 1d). However, we found no significant differences in KEGG pathway functional genes between intestinal flora and cognitive function in MHD patients.

## Discussion

Due to the limitations of experimental funds and difficulties in collecting clinical data, there is no uniform standard for determining an appropriate sample size. Furthermore, the traditional formula for sample size estimation is not very applicable. Therefore, we referred to literature exploring the role of gut microbiota in disease models, such as a study of 41 pre-dialysis diabetic kidney disease patients [19] and 51 end-stage renal disease patients [20]. On this basis, we selected an appropriate sample size for our studies.

Cerebrovascular disease, age, gender, and education level are important factors affecting cognitive dysfunction [21, 22]; hence, all people involved in our experiment were free from cerebrovascular disease, and both groups were recruited by matching age, sex, and education to avoid potential confounding factors, and to discover other factors affecting cognitive function. We applied the MoCA test to assess cognitive function because it is rapid, comprehensive, and widely applied. The mean age of maintenance hemodialysis (MHD) patients was 61.06 years, the Montreal score was 25 points, and the prevalence of cognitive impairment was 61%. The Montreal score of MHD patients was significantly lower than that of the healthy group, mainly in terms of visuospatial/executive function, naming, and language. The results of prevalence and the specific domains of cognitive impairment among MHD patients were consistent with previous studies [5–8]. However, the median scores for our cohort were slightly higher than in previous studies [6].

The Chao1 index was found to be decreased in intestinal flora of end-stage of renal disease (ESRD) patients in southern China [20]. While, our results revealed increased biodiversity in MHD patients, as evidenced by an increase in Chao1 index and Observed_species index. Analyzing the reasons for this phenomenon in our study, we believe that one reason is that the intestinal flora is affected by many factors, such as diet, region, and some confounding factors. Compared with the southern study, the subjects participating in our study are all from northern China and have started hemodialysis treatment. We also have more participants in our experiment. These different factors may explain the different results between our results and those of Jiang et al. (2017).

In addition, our Shannon index shows that there is no difference in microbial diversity between the MHD group and the healthy group, which is consistent with those of Noriaki et al. [23] and Wu et al. [24]. The difference between Shannon and Chao1 index as evaluating microbial diversity tools in bacterial diversity is that, in addition to being consistent with Chao1’s role in evaluating species richness, Shannon also considers community evenness. Combining the high species richness of the MHD patients in our study and the no difference in Shannon index, we infer that the community evenness of the MHD is poor, which is consistent with the significant intra-group differences shown in our Fig. 2d. 

The relative abundance and statistical differences in the relative abundance were analyzed separately in 16S rDNA and shotgun metagenomic sequencing. Both sets of results showed that *Firmicutes*, *Bacteroidetes*, *Proteobacteria*, and *Actinobacteria* were the main components of the microbiota. These results are consistent with those of previous research on Western and Asian countries [20, 26]. We were also surprised to observe a significant reduction in *Roseburia* in MHD patients. A reduction of *Roseburia* has been reported previously in studies on patients with early-stage CKD [23] and end-stage renal disease [19]. *Roseburia* members are likely to be beneficial bacteria due to their anti-inflammatory effects and ability to modulate sugar and protein metabolism [27]. *Roseburia* bind to Toll-like receptor 5 on intestinal epithelium cells through the flagellum, stimulating dendritic cells to secrete cytokines and promoting differentiation of regulatory T cells, thereby exerting anti-inflammatory effects. Also, butyrate produced by *Roseburia* binds to G-protein-coupled receptors and participates in the regulation of glycoprotein metabolism pathways. Wang et al. [28] identified two major enterotypes (*Faecalibacterium prausnitzii* and *Prevotella*) and *Roseburia* were associated with reduced production of short-chain fatty acids. Thus, this microbial imbalance further aggravates inflammatory responses and promotes the progression of kidney disease. In the present work, *Blautia*, *Erysipelatoclostridium*, *Phascolarctobacterium*, *Sellimonas*, and *Butyricicoccus* were identified, all of which can produce short-chain fatty acids (SCFAs) [29], which indicates that SCFAs are also involved in the progression of advanced CKD. In addition, such differential bacterial flora may provide a potential therapeutic strategy for CKD management through supplementation of SCFAs combined with selected prebiotics or probiotics.

Based on correlation analysis of cognitive function and intestinal flora between dialysis patients and healthy people, *Faecalibacterium*, *Megamonas*, *Bifidobacterium*, *Parabacteroides*, *Collinsella*, *Tyzzerella*, and *Phascolarctobacterium* were positively correlated with cognitive function or cognitive domains. This may provide evidence for the gut–brain axis modulating cognitive function, even in relatively young patients with stage 5 CKD. A brain–gut–kidney axis has been proposed involving neural, immune, and metabolic interactions [30]. After the brain is stimulated by factors such as the environment, diet, and CKD-related stimulatory factors, it activates the sympathetic nervous system throughout the body. On the one hand, the sympathetic nervous system promotes the activation of immune cells in bone marrow, leading to an increase in the level of inflammation; on the other hand, it also alters the permeability of the intestines, allowing intestinal bacteria and increased intestinal flora metabolites to participate in blood circulation. Eventually, excessive activation of inflammation and accumulation of toxic metabolites promote the progression of kidney disease [30].

*F. prausnitzii*, one of the most important bacteria in the human intestinal flora, plays a beneficial probiotic role in intestinal physiology and maintains host health by producing butyric acid via G-protein-coupled receptor 43 Axis [31], and accounts for 5–15% of total bacteria detected in healthy human stool samples [32]. A study on healthy Dutch adults (65–79 years old) exploring the relationships between diet, gastrointestinal microbiota composition, and cognitive function found that *F. prausnitzii* and *Roseburia* exhibit anti-inflammatory properties and are associated with beneficial health effects [33]. Herein, we were not surprised to observe a correlation between *Bifidobacteria* and cognitive function since numerous intervention studies found that *Bifidobacteria* supplementation can improve cognitive function [34, 35]. We are very interested in *Phascolarctobacterium* because members were both differentially expressed in MHD patients, and correlated with cognitive function. Although the exact mechanisms through which *Phascolarctobacterium* affects cognitive function are not known, members play a key role in various disease models. In autism spectrum disorder, *Phascolarctobacterium* are increased in abundance and their metabolites may contribute to the occurrence and severity of the disorder [36]. *Phascolarctobacterium* is involved in intestinal flora processes, neuroactive metabolites, and brain functional connectivity networks in bipolar depression [37]. In any case, these intestinal floras provide evidence for a functional gut–brain axis, and a potential treatment strategy for alleviating cognitive dysfunction in patients.

Our functional gene annotation based on shotgun metagenomic sequencing results identified metabolic pathways that maybe related to the occurrence of cognitive disorder. Lipopolysaccharide biosynthesis is enriched in the microbiome at the advanced stage of CKD [25], and oxidative phosphorylation affects cognitive function by causing the accumulation of iron complexes [38]. The retrograde endocannabinoid signaling pathway is essential for core cognitive processes in schizophrenia, such as working memory [39] and involved in the regulation of anxiety and depression-like behaviors [40]. Riboflavin metabolism is linked to vitamin B, and vitamin B deficiency is associated with neurocognitive disorders [41].

Herein, we performed a simple metagenomic difference analysis between HD.UN and HD.N to explore links between intestinal flora and cognitive function. We found that *Megamonas_funiformis* and *Akkermansiaceae* of *Verrucomicrobiales* were more abundant in the HD.UN group. *Megamonas_funiformis* plays a pivotal role in the progression of colorectal cancer (CRC) [42]. *Verrucomicrobiales* is associated with inflammatory activity in patients with non-alcoholic steatohepatitis (NASH) without metabolic syndrome (MS) [43]. However, we failed to find any meaningful KEGG pathway functional genes linking intestinal flora and cognitive function in MHD patients. This may be due to the limited sample size, which may affect our ability to identify metabolic pathways of intestinal flora involved in the process of cognitive dysfunction. In the future, we will increase the sample size of HD-UN and HD-N to explore the underlying mechanisms linking intestinal flora and cognitive function.

There are some shortcomings with our study. First, the sample size was relatively small. Although we accounted for the effects of some known factors, including age and education, we cannot avoid the interference of some uncertain factors. We only explored the correlation between intestinal flora and cognitive function in dialysis patients, but not CKD patients at different stages. We predicted functional genes of metabolic pathways via which the intestinal flora may affect cognitive function, but the results require validation in vivo.

In summary, we carried out a detailed exploration of the cognitive functions of MHD patients, and investigated potential links between changes in the intestinal microflora and cognitive function in MHD patients. By combining 16SrDNA and shotgun metagenomic sequencing, we found a correlation between changes in intestinal flora and cognitive function in MHD patients, and propose a possible mechanism for the first time.

### Supplementary Information

Below is the link to the electronic supplementary material.

Supplementary file1 (DOCX 228 KB)

## Data Availability

The data used in this article come from the author's own collection and analysis of data. The sequencing data used in this study have been uploaded to the Internet and are publicly accessible. Specific URLs of the data are given in the Materials and Methods section. The format and content of the data are consistent with the analyzes and results presented in this article. We guarantee the integrity and accuracy of the data and are responsible for the quality of the data.
