# Supplementary material for: Analysis of intestinal flora and cognitive function in maintenance hemodialysis patients using combined 16S ribosome DNA and shotgun metagenome sequencing
Source: Aging Clin Exp Res. 2024 Feb 9;36(1):28. doi: 10.1007/s40520-023-02645-y (PMC10857965; doi:10.1007/s40520-023-02645-y)
Supplement: Supplementary file 1 — Supplementary file1 (DOCX 228 KB) [file 40520_2023_2645_MOESM1_ESM.docx]

***Supplementary Material***

**Supplementary Data**

**1 Details for 16s rDNA amplifiers and Metagenomic sequencing**

**1.1 Material method of 16s rDNA Sequencing**

**1.1.1 Extraction of genome DNA Total genome**

DNA from samples was extracted using CTAB/SDS method. DNA concentration and purity was monitored on 1% agarose gels. According to the concentration, DNA was diluted to 1 ng/µL using sterile water.

**1.1.2 Amplicon Generation**

16S rRNA genes were amplified used specific primer 16S V4: 515F-806R with the barcode. All PCR reactions were carried out in 30 µL reactions with 15 µL of Phusion® High-Fidelity PCR Master Mix (New England Biolabs); 0.2 µM of forward and reverse primers, and about 10ng template DNA. Thermal cycling consisted of initial denaturation at 98℃for 1 min, followed by 30 cycles of denaturation at 98℃for 10 s, annealing at 50℃for 30 s, and elongation at 72℃ for 30 s. Finally 72℃for 5 min.

**1.1.3 PCR**

Products Mixing and Purification Mix same volume of 1×loading buffer (contained SYB green) with PCR products and operate electrophoresis on 2% agarose gel for detection. PCR products was mixed in equidensity ratios. Then, mixture PCR products was purified with GeneJETTM Gel Extraction Kit (Thermo Scientific).

**1.1.4 Library preparation and sequencing**

Sequencing libraries were generated using Ion Plus Fragment Library Kit 48 rxns (Thermo Scientific) following manufacturer's recommendations. The library quality was assessed on the Qubit@ 2.0 Fluorometer (Thermo Scientific). At last, the library was sequenced on an Ion S5TM XL platform and 400 bp/600 bp single-end reads were generated.

**1.1.5 16s information analysis**

The original data was cut by Cutadapt (V1.9.1)([1](#_ENREF_1)) and filtered to get the effective data (CleanData). Then all valid data of all samples are clustered into Operational taxonomic units (OTUs) with 97% consistency by using Uparse (Uparse v7.0.1001)([2](#_ENREF_2)). the OTUs sequence was compared with the SSUrRNA([3](#_ENREF_3)) database of SILVA132 ([4](#_ENREF_4)) by Mothur method to obtain the species annotation information at each classification level (threshold is 0.8-1). Qiime software (Version1.9.1) was used to calculate Observed-otus, Chao1, Shannon, Simpson index to evaluate the sample complexity analysis (Alpha Diversity). While the AD4 package and ggplot2 package of R software (Version2.15.3) were used to draw PCA diagram to analyze the difference of Beta diversity index among groups. PCA uses the method of variance decomposition to reduce the multi-dimensional data into two-dimensional data, and reflect the difference in a two-dimensional coordinate graph ([5](#_ENREF_5)).

**1.2 Material method of Metagenomic sequencing：**

**1.2.1 Quality Control for DNA samples:**

Extracted DNA of samples were qualitatively measured by 1% agarose gel and quantitatively measured by Qubit® dsDNA Assay Kit in Qubit® 2.0 Flurometer (Life Technologies, CA, USA), those concentration greater than 1ug was used to construct library.

**1.2.1 Library construction**

A total amount of 1μg DNA per sample was used as input material for the DNA sample preparations. Sequencing libraries were generated using NEBNext® Ultra™ DNA Library Prep Kit for Illumina (NEB, USA) following manufacturer’s recommendations and index codes were added to attribute sequences to each sample. Briefly, the qualified DNA sample was fragmented by sonication to a size of 350bp,then DNA fragments were end-polished, A-tailed, and ligated with the full-length adaptor for Illumina sequencing with further PCR amplification. At last, PCR products were purified (AMPure XP system) and libraries were analysed for size distribution by Agilent2100 Bioanalyzer and quantified using real-timePCR.

**1.2.3 Sequencing**

The clustering of the index-coded samples was performed on a cBot Cluster Generation System according to the manufacturer’s instructions. After cluster generation, the library preparations were sequenced on an Illumina HiSeq platform and paired-end reads were generated.

**1.2.4 Analysis of metagenomic sequencing**

**1.2.4.1 Gene prediction and abundance analysis**

**1)**The Scaftigs (≥ 500 bp) assembled from both single and mixed are all predicted the ORF by MetaGeneMark (V2.10, http://topaz.gatech.edu/GeneMark/) software, and filtered the length information shorter than 100 nt from the predicted result with default parameters.

**2)** For ORF predicted, CD-HIT software (V4.5.8, http://www.bioinformatics.org/cd-hit ) is adopted to redundancy and obtain the unique initial gene catalogue (the genes here refers to the nucleotide sequences coded by unique and continuous genes([6](#_ENREF_6))), the parameters option are -c 0.95, -G 0, -aS 0.9, -g 1, -d 0.

**3)** The Clean Data of each sample is mapped to initial gene catalogue using Bowtie2.2.4 and get the number of reads to which genes mapped in each sample with the parameter setting are -- end-to-end, --sensitive, -I 200, -X 400. Filter the gene which the number of reads ≤ 2 in each sample and obtain the gene catalogue (Unigenes) eventually used for subsequently analysis.

**4)**Based on the number of mapped reads and the length of gene, statistic the abundance information of each gene in each sample. The format is as follow, r represents the number of reads mapped to the genes and L represents gene’s length.

**5)**The basic information statistic, core-pan gene analysis, correlation analysis of samples and venn figure analysis of number of genes are all based on the abundance of each gene in each sample in gene catalogue.

**1.2.4.2 Taxonomy prediction**

**1)** DIAMOND software (V0.9.9, https://github.com/bbuchfink/diamond/) is used to blast the Unigenes to the sequences of Bacteria, Fungi, Archaea and Viruses which are all extracted from the NR database (Version: 2018-01-02, https://www.ncbi.nlm.nih.gov/) of NCBI with the parameter setting are blastp-e 1e-5.

**2)**For the finally aligned results of each sequence, as each sequence may have multiple aligned results, choose the result of which the e value ≤ the smallest e value * 10 to take the LCA algorithm which is applied to system classification of MEGAN software to make sure the species annotation information of sequences.

**3)** The table containing the number of genes and the abundance information of each sample in each taxonomy hierarchy (kingdom, phylum, class, order, family, genus, species) are obtained based on the LCA annotation result and the gene abundance table. The abundance of a specie in one sample equal the sum of the gene abundance annotated for the specie; the gene number of a specie in a sample equal the number of genes whose abundance are nonzero.

**1.2.4.3 Common functional database annotations**

**1)** Adopt DIAMOND software (V0.9.9) to blast Unigenes to functional database with the parameter setting of blastp, -e 1e-5. Functional database exclude KEGG database (Version 2018-01-01, http://www.kegg.jp/kegg/), eggNOG database (Version 4.5, http://eggnogdb.embl.de/#/app/home), CAZy database (Version 201801, http://www.cazy.org/). For each sequence’s blast result, the best Blast Hit is used for subsequent analysis.

**2)** Statistic of the relative abundance of different functional hierarchy, the relative abundance of each functional hierarchy equal the sum of relative abundance annotated to that functional level.

**3)** Based on the function annotation result and gene abundance table, the gene number table of each sample in each taxonomy hierarchy is obtained. The gene number of a function in a sample equal the gene number that annotated to this function and the abundance is nonzero.

**4)** Based on the abundance table of each taxonomy hierarchy, not only the counting of annotated gene numbers, the exhibition of the general relative abundance situation, the exhibition of abundance cluster heat map and the decrease-dimension analysis of PCA are conducted, the LEfSe analysis of functional difference between groups are performed.

**2 Supplementary Figure 1**

Supplementary Figure 1 (a, b, c) Differences in α-diversity result between MHD with (MHD.UN) and without (MHD.N) cognitive disorder based on metagenomic sequencing. Chao1, Observed_species and Shannon indices show a significant increase in MHD.N patients.(d) Differences in the intestinal microbiota between MHD.UN and MHD.N. S_ Megamonas_ funiformis, 0_ Verrucomicrobiales and f_Akkermansiaceae were more abundant in the HD.UN group. S_ Sutterella_ wadsworthensis, s_ Bacteroidales_ bacterium_ 43_ 36, g_ Chryseobacterium, S_ Azobacteroides_phage_ PrpJPt_ Bp1, S__ Porphyromonadaceae_ bacterium_ KH3CP3RA and g_ Sutterella, S_ Veillonella_ sp_ CAG_ 933 were more abundant in the HD. N group.

Supplement Figure 1 Differences in α-diversity result between MHD with (MHD.UN) and without (MHD.N) cognitive disorder based on metagenomic sequencing


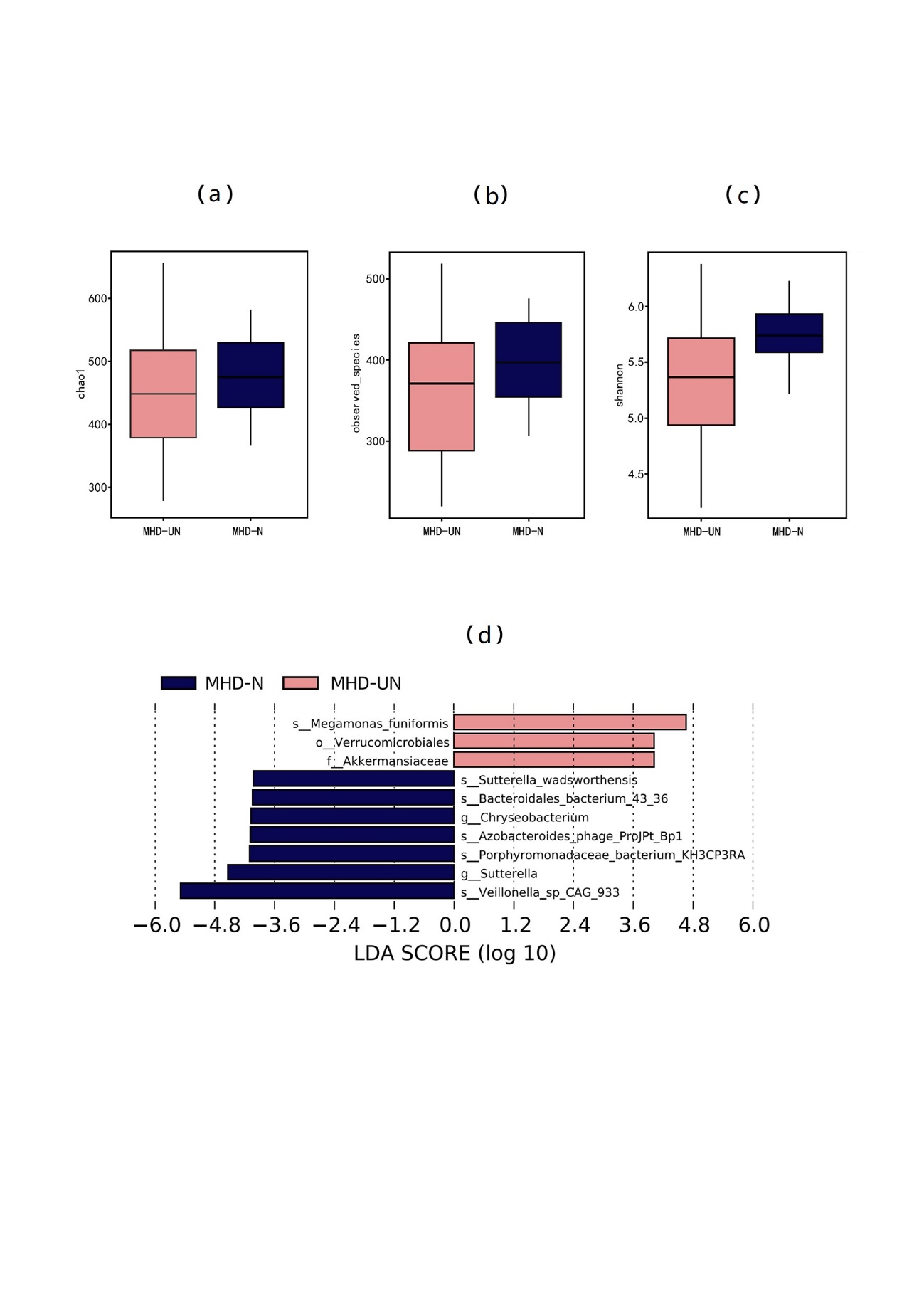


**3 Reference**

1. Kechin A, Boyarskikh U, Kel A, Filipenko M (2017) cutPrimers: A New Tool for Accurate Cutting of Primers from Reads of Targeted Next Generation Sequencing. Journal of computational biology : a journal of computational molecular cell biology 24:1138-1143. https://doi.org/10.1089/cmb.2017.0096.

2. Rognes T, Flouri T, Nichols B, Quince C, Mahé F (2016) VSEARCH: a versatile open source tool for metagenomics. PeerJ 4:e2584. https://doi.org/10.7717/peerj.2584.

3. Edgar RC (2013) UPARSE: highly accurate OTU sequences from microbial amplicon reads. Nature methods 10:996-998. https://doi.org/10.1038/nmeth.2604.

4. Haas BJ, Gevers D, Earl AM, Feldgarden M, Ward DV, Giannoukos G, et al (2011) Chimeric 16S rRNA sequence formation and detection in Sanger and 454-pyrosequenced PCR amplicons. Genome research 21:494-504. https://doi.org/10.1101/gr.112730.110

5. Tiffin-Richards FE, Costa AS, Holschbach B, Frank RD, Vassiliadou A, Kruger T, et al (2014) The Montreal Cognitive Assessment (MoCA) - a sensitive screening instrument for detecting cognitive impairment in chronic hemodialysis patients. PloS one 9:e106700. https://doi.org/ 10.1371/journal.pone.0106700

6. Sunagawa S, Coelho LP, Chaffron S, Kultima JR, Labadie K, Salazar G, et al (2015) Ocean plankton. Structure and function of the global ocean microbiome. Science (New York, NY). 348(6237):1261359. https://doi.org/ 10.1126/science.1261359
